# *N*,*N*′-Dicyclo­hexyl-*N*′′-(3-fluoro­benzo­yl)-*N*,*N*′-dimethyl­phospho­ric triamide

**DOI:** 10.1107/S1600536811043017

**Published:** 2011-10-22

**Authors:** Mehrdad Pourayoubi, Samad Shoghpour, Giuseppe Bruno, Hadi Amiri Rudbari

**Affiliations:** a Department of Chemistry, Ferdowsi University of Mashhad, Mashhad 91779, Iran; b Dipartimento di Chimica Inorganica, Vill. S. Agata, Salita Sperone 31, Università di Messina, 98166 Messina, Italy

## Abstract

In the title compound, C_21_H_33_FN_3_O_2_P, the P atom has a distorted tetra­hedral environment and the N atoms display geometries consistent with a model of *sp* ^2^ hybridization (with bond-angle sums for the tertiary N atoms of 357.8 and 358.7°). The phosphoryl and carbonyl groups are *anti* with respect to each other. In the crystal, inversion dimers linked by pairs of N—H⋯O hydrogen bonds generate *R*_2_^2^(8) loops.

## Related literature

For the coordination properties of carbacyl­amido­phosphates, see: Pourayoubi *et al.* (2011*b* ▶); Gholivand *et al.* (2010 ▶); Znovjyak *et al.* (2009 ▶); Trush *et al.* (2005 ▶); Gubina *et al.* (2002 ▶). For related structures, see: Pourayoubi *et al.* (2011*a* ▶); Pourayoubi & Saneei (2011 ▶). For the *syn* orientation of the P(=O) group and NH unit in the C(O)NHP(O) skeleton for most known carbacyl­amido­phosphates, see: Toghraee *et al.* (2011 ▶). For a procedure to synthesise the starting phospho­rus–chlorine compound, see: Pourayoubi *et al.* (2011*c* ▶). For graph-set notation of hydrogen bonds, see: Bernstein *et al.* (1995 ▶).
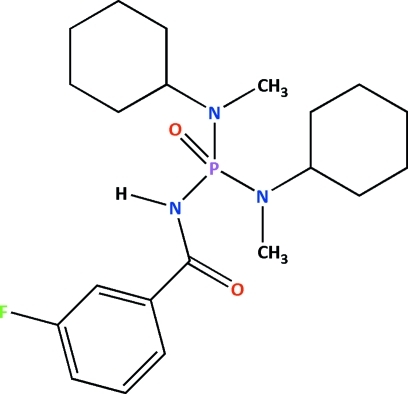


## Experimental

### 

#### Crystal data

C_21_H_33_FN_3_O_2_P
*M* *_r_* = 409.47
Monoclinic, 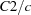

*a* = 22.6634 (8) Å
*b* = 12.9587 (5) Å
*c* = 17.6627 (7) Å
β = 119.061 (1)°
*V* = 4534.3 (3) Å^3^
*Z* = 8
Mo *K*α radiation
μ = 0.15 mm^−1^
*T* = 296 K
0.32 × 0.28 × 0.16 mm

#### Data collection

Bruker APEXII CCD diffractometer
Absorption correction: multi-scan (*SADABS*; Sheldrick, 2008 ▶) *T*_min_ = 0.658, *T*_max_ = 0.746
20958 measured reflections
4228 independent reflections
3254 reflections with *I* > 2σ(*I*)
*R*_int_ = 0.031

#### Refinement

*R*[*F* ^2^ > 2σ(*F* ^2^)] = 0.049
*wR*(*F* ^2^) = 0.157
*S* = 1.02
4228 reflections
257 parameters
1 restraint
H atoms treated by a mixture of independent and constrained refinement
Δρ_max_ = 0.93 e Å^−3^
Δρ_min_ = −0.29 e Å^−3^

### 

Data collection: *APEX2* (Bruker, 2007 ▶); cell refinement: *SAINT* (Bruker, 2007 ▶); data reduction: *SAINT*; program(s) used to solve structure: *SHELXS97* (Sheldrick, 2008 ▶); program(s) used to refine structure: *SHELXL97* (Sheldrick, 2008 ▶); molecular graphics: *OLEX* (Dolomanov *et al.*, 2003 ▶); software used to prepare material for publication: *SHELXTL* (Sheldrick, 2008 ▶) and *enCIFer* (Allen *et al.*, 2004 ▶).

## Supplementary Material

Crystal structure: contains datablock(s) I, global. DOI: 10.1107/S1600536811043017/qm2034sup1.cif

Structure factors: contains datablock(s) I. DOI: 10.1107/S1600536811043017/qm2034Isup2.hkl

Additional supplementary materials:  crystallographic information; 3D view; checkCIF report

## Figures and Tables

**Table 1:** Hydrogen-bond geometry (Å, °)

*D*—H⋯*A*	*D*—H	H⋯*A*	*D*⋯*A*	*D*—H⋯*A*
N1—H1⋯O1^i^	0.78 (2)	2.04 (2)	2.807 (2)	165 (2)

Symmetry code: (i) 
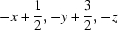
.

**Fig. 1.**
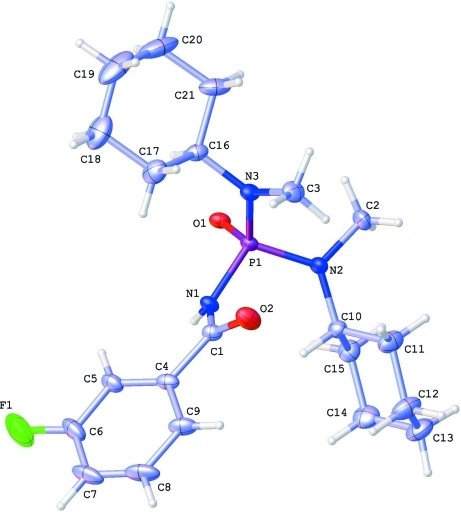
An ORTEP-style plot of title compound with labeling. Ellipsoids are given at the 50% probability level.

## supplementary crystallographic information

### Comment

It is now well recognized that carbacylamidophosphates offer very good candidates for coordination chemistry purposes, since they bear a C(O)NHP(O) bifunctional group which is the phosphaza- analogue of β-diketones. In this context, a variety of coordination compounds with both transition and non-transition metal cations have been reported (Pourayoubi *et al.*, 2011*b*; Gholivand *et al.*, 2010; Znovjyak *et al.*, 2009; Trush *et al.*, 2005; Gubina *et al.*, 2002). Therefore, the synthesis and crystal structure investigations of carbacylamidophosphates have been of particular interest in our research team (Pourayoubi *et al.*, 2011*a*; Pourayoubi & Saneei, 2011). In this work, the synthesis and crystal structure of a new carbacylamidophosphate, P(O)[NHC(O)C_6_H_4_(3-F)][N(CH_3_)(C_6_H_11_)]_2_, is reported. The molecular structure (*ORTEP* view) of the title compound is shown in Fig. 1. The phosphoryl group and the NH unit are located in a *syn* position with respect to each other similar to most of the carbacylamidophosphates (Toghraee *et al.*, 2011). The P atom has a distorted tetrahedral configuration with the bond angles around the P atom in the range of 105.9 (1)–116.4 (1)°. The P═O, C═O and P—N bond lengths and the P—N—C bond angles are in the range of the expected values. The sum of the surrounding angles around the tertiary N atoms confirms their *sp*^2^ hybridization. In the crystal structure, two neighboring molecules are hydrogen-bonded to each other by two equal intermolecular P═O···H—N hydrogen bonds (O1···N1 = 2.807 (2) Å) (Table 1) to form a centrosymmetric dimer as an *R*_2_^2^(8) ring.

### Experimental

Synthesis of 3-F–C_6_H_4_C(O)NHP(O)Cl_2_ 3-F–C_6_H_4_C(O)NHP(O)Cl_2_ was prepared according to the procedure which was previously used for preparation of 2-F–C_6_H_4_C(O)NHP(O)Cl_2_ (Pourayoubi *et al.*, 2011*c*) by using 3-F–C_6_H_4_C(O)NH_2_ instead of 2-F–C_6_H_4_C(O)NH_2_. Synthesis of the title molecule To a solution of 3-F–C_6_H_4_C(O)NHP(O)Cl_2_ (0.512 g, 2 mmol) in CHCl_3_ (20 ml), a solution of *N*-methylcyclohexylamine (0.906 g, 8 mmol) in CHCl_3_ (5 ml) was added dropwise at 273 K. After 4 h stirring, the solvent was evaporated *in vacuo* and then the resulting solid was washed with water. Single crystals of title compound were obtained from a solution of CH_3_OH and CHCl_3_ (2:1) after slow evaporation at room temperature. IR (KBr, cm^-1^): 3068 (NH), 2930, 2855, 1685 (C═O), 1589, 1491, 1448, 1393, 1287, 1272, 1181, 1161, 1005, 982, 886, 860, 749, 682.

### Refinement

Several H atoms were located on the final ΔF map, the H atoms were included in the refinement using the `riding model' method with the *X*—H bond geometry and the H isotropic displacement parameter depending on the parent atom *X*.

### Crystal data

**Table:**

C_21_H_33_FN_3_O_2_P	*F*(000) = 1760
*M**_r_* = 409.47	*D*_x_ = 1.200 Mg m^−^^3^
Monoclinic, *C*2/*c*	Mo *K*α radiation, λ = 0.71073 Å
Hall symbol: -C 2yc	Cell parameters from 8133 reflections
*a* = 22.6634 (8) Å	θ = 2.4–27.0°
*b* = 12.9587 (5) Å	µ = 0.15 mm^−^^1^
*c* = 17.6627 (7) Å	*T* = 296 K
β = 119.061 (1)°	Cubic, colourless
*V* = 4534.3 (3) Å^3^	0.32 × 0.28 × 0.16 mm
*Z* = 8	

### Data collection

**Table:**

Bruker APEXII CCD diffractometer	4228 independent reflections
Radiation source: fine-focus sealed tube	3254 reflections with *I* > 2σ(*I*)
graphite	*R*_int_ = 0.031
φ and ω scans	θ_max_ = 25.5°, θ_min_ = 2.6°
Absorption correction: multi-scan (*SADABS*; Sheldrick, 2008)	*h* = −27→27
*T*_min_ = 0.658, *T*_max_ = 0.746	*k* = −15→15
20958 measured reflections	*l* = −21→21

### Refinement

**Table:**

Refinement on *F*^2^	Primary atom site location: structure-invariant direct methods
Least-squares matrix: full	Secondary atom site location: difference Fourier map
*R*[*F*^2^ > 2σ(*F*^2^)] = 0.049	Hydrogen site location: inferred from neighbouring sites
*wR*(*F*^2^) = 0.157	H atoms treated by a mixture of independent and constrained refinement
*S* = 1.02	*w* = 1/[σ^2^(*F*_o_^2^) + (0.0936*P*)^2^ + 3.0653*P*] where *P* = (*F*_o_^2^ + 2*F*_c_^2^)/3
4228 reflections	(Δ/σ)_max_ < 0.001
257 parameters	Δρ_max_ = 0.93 e Å^−^^3^
1 restraint	Δρ_min_ = −0.29 e Å^−^^3^

### Special details

**Table:**

Geometry. All s.u.'s (except the s.u. in the dihedral angle between two l.s. planes) are estimated using the full covariance matrix. The cell s.u.'s are taken into account individually in the estimation of s.u.'s in distances, angles and torsion angles; correlations between s.u.'s in cell parameters are only used when they are defined by crystal symmetry. An approximate (isotropic) treatment of cell s.u.'s is used for estimating s.u.'s involving l.s. planes.
Refinement. Refinement of *F*^2^ against ALL reflections. The weighted *R*-factor *wR* and goodness of fit *S* are based on *F*^2^, conventional *R*-factors *R* are based on *F*, with *F* set to zero for negative *F*^2^. The threshold expression of *F*^2^ > 2σ(*F*^2^) is used only for calculating *R*-factors(gt) *etc*. and is not relevant to the choice of reflections for refinement. *R*-factors based on *F*^2^ are statistically about twice as large as those based on *F*, and *R*-factors based on ALL data will be even larger.

### Fractional atomic coordinates and isotropic or equivalent isotropic displacement parameters (Å^2^)

**Table:**

	*x*	*y*	*z*	*U*_iso_*/*U*_eq_	
P1	0.19691 (2)	0.87686 (4)	0.02588 (3)	0.03552 (19)	
O2	0.30019 (9)	0.98806 (14)	0.18782 (12)	0.0637 (5)	
F1	0.46037 (14)	0.6077 (2)	0.31270 (17)	0.1416 (10)	
O1	0.16844 (7)	0.78142 (11)	−0.02507 (9)	0.0426 (4)	
N1	0.27588 (8)	0.84697 (15)	0.10211 (11)	0.0370 (4)	
C1	0.31644 (10)	0.90341 (18)	0.17448 (13)	0.0417 (5)	
C3	0.15054 (14)	1.02308 (19)	0.0955 (2)	0.0618 (7)	
H3A	0.1710	1.0669	0.0709	0.093*	
H3B	0.1751	1.0282	0.1575	0.093*	
H3C	0.1047	1.0443	0.0749	0.093*	
N2	0.20318 (9)	0.97566 (15)	−0.02662 (12)	0.0479 (5)	
C19	0.0611 (3)	0.7585 (4)	0.2030 (4)	0.135 (2)	
H19A	0.0582	0.8200	0.2323	0.162*	
H19B	0.0428	0.7012	0.2204	0.162*	
C20	0.0200 (2)	0.7734 (4)	0.1057 (4)	0.1268 (18)	
H20A	−0.0261	0.7903	0.0905	0.152*	
H20B	0.0193	0.7094	0.0769	0.152*	
C21	0.04874 (15)	0.8595 (3)	0.0735 (2)	0.0828 (10)	
H21A	0.0231	0.8637	0.0108	0.099*	
H21B	0.0450	0.9252	0.0971	0.099*	
C16	0.12210 (11)	0.83718 (19)	0.10158 (15)	0.0477 (5)	
H16	0.1235	0.7713	0.0752	0.057*	
N3	0.15171 (9)	0.91587 (14)	0.06955 (12)	0.0429 (4)	
C4	0.38187 (11)	0.8550 (2)	0.23687 (13)	0.0458 (6)	
C5	0.39071 (12)	0.7492 (2)	0.24479 (15)	0.0563 (6)	
H5	0.3560	0.7044	0.2099	0.068*	
C6	0.45265 (15)	0.7122 (3)	0.30609 (18)	0.0740 (9)	
C7	0.50556 (14)	0.7743 (4)	0.35762 (17)	0.0865 (12)	
H7	0.5470	0.7466	0.3975	0.104*	
C18	0.1330 (3)	0.7373 (3)	0.2291 (3)	0.1109 (15)	
H18A	0.1362	0.6723	0.2041	0.133*	
H18B	0.1588	0.7311	0.2917	0.133*	
C17	0.16264 (17)	0.8231 (3)	0.19916 (18)	0.0774 (9)	
H17A	0.1626	0.8871	0.2277	0.093*	
H17B	0.2090	0.8064	0.2154	0.093*	
C10	0.26677 (11)	1.00986 (18)	−0.02259 (15)	0.0462 (5)	
H10	0.3035	0.9781	0.0293	0.055*	
C15	0.27532 (14)	0.9741 (2)	−0.09852 (16)	0.0599 (7)	
H15A	0.2726	0.8994	−0.1022	0.072*	
H15B	0.2390	1.0019	−0.1518	0.072*	
C14	0.34258 (17)	1.0089 (3)	−0.0885 (2)	0.0776 (9)	
H14A	0.3454	0.9893	−0.1397	0.093*	
H14B	0.3788	0.9743	−0.0391	0.093*	
C13	0.35163 (19)	1.1247 (3)	−0.0759 (2)	0.0881 (10)	
H13A	0.3961	1.1439	−0.0664	0.106*	
H13B	0.3183	1.1595	−0.1277	0.106*	
C12	0.34381 (19)	1.1582 (3)	0.0008 (3)	0.0898 (10)	
H12A	0.3480	1.2326	0.0066	0.108*	
H12B	0.3796	1.1278	0.0532	0.108*	
C11	0.27582 (17)	1.1256 (2)	−0.0098 (2)	0.0723 (8)	
H11A	0.2729	1.1454	0.0412	0.087*	
H11B	0.2400	1.1606	−0.0594	0.087*	
C2	0.13971 (13)	1.0138 (2)	−0.09931 (19)	0.0692 (8)	
H2A	0.1022	0.9864	−0.0948	0.104*	
H2B	0.1367	0.9921	−0.1530	0.104*	
H2C	0.1389	1.0878	−0.0975	0.104*	
C9	0.43442 (12)	0.9204 (3)	0.28957 (16)	0.0652 (7)	
H9	0.4284	0.9915	0.2851	0.078*	
C8	0.49597 (15)	0.8784 (4)	0.34893 (19)	0.0887 (12)	
H8	0.5315	0.9221	0.3836	0.106*	
H1	0.2925 (11)	0.8035 (17)	0.0877 (14)	0.038 (6)*	

### Atomic displacement parameters (Å^2^)

**Table:**

	*U*^11^	*U*^22^	*U*^33^	*U*^12^	*U*^13^	*U*^23^
P1	0.0267 (3)	0.0428 (3)	0.0322 (3)	0.0018 (2)	0.0104 (2)	−0.0015 (2)
O2	0.0569 (11)	0.0588 (11)	0.0590 (11)	0.0018 (8)	0.0153 (9)	−0.0221 (8)
F1	0.117 (2)	0.160 (2)	0.1197 (19)	0.0660 (17)	0.0356 (16)	0.0554 (17)
O1	0.0301 (7)	0.0530 (9)	0.0395 (8)	−0.0025 (6)	0.0128 (6)	−0.0097 (7)
N1	0.0297 (9)	0.0432 (10)	0.0319 (9)	0.0053 (7)	0.0100 (7)	−0.0046 (7)
C1	0.0344 (11)	0.0534 (14)	0.0345 (11)	−0.0035 (9)	0.0145 (9)	−0.0065 (9)
C3	0.0623 (16)	0.0495 (15)	0.0805 (19)	0.0079 (12)	0.0401 (15)	−0.0068 (13)
N2	0.0341 (10)	0.0566 (12)	0.0448 (10)	0.0024 (8)	0.0127 (8)	0.0117 (9)
C19	0.197 (6)	0.116 (3)	0.183 (5)	−0.055 (3)	0.164 (5)	−0.036 (3)
C20	0.098 (3)	0.134 (4)	0.195 (5)	−0.058 (3)	0.108 (4)	−0.058 (4)
C21	0.0469 (15)	0.108 (2)	0.099 (2)	−0.0154 (15)	0.0406 (16)	−0.0275 (19)
C16	0.0438 (12)	0.0544 (14)	0.0504 (13)	−0.0089 (10)	0.0272 (11)	−0.0132 (11)
N3	0.0382 (10)	0.0433 (10)	0.0495 (10)	0.0031 (8)	0.0231 (8)	−0.0041 (8)
C4	0.0318 (11)	0.0755 (17)	0.0283 (10)	−0.0034 (10)	0.0133 (9)	−0.0064 (10)
C5	0.0392 (13)	0.0812 (19)	0.0388 (12)	0.0100 (12)	0.0113 (10)	0.0077 (12)
C6	0.0586 (17)	0.106 (2)	0.0518 (15)	0.0363 (17)	0.0225 (14)	0.0271 (16)
C7	0.0364 (15)	0.173 (4)	0.0375 (14)	0.0246 (19)	0.0079 (12)	0.0137 (19)
C18	0.168 (5)	0.103 (3)	0.096 (3)	−0.021 (3)	0.090 (3)	0.008 (2)
C17	0.080 (2)	0.096 (2)	0.0561 (17)	−0.0145 (17)	0.0327 (16)	0.0047 (16)
C10	0.0408 (12)	0.0554 (14)	0.0414 (12)	−0.0026 (10)	0.0192 (10)	0.0040 (10)
C15	0.0671 (17)	0.0661 (17)	0.0508 (14)	−0.0006 (13)	0.0321 (13)	−0.0005 (12)
C14	0.081 (2)	0.104 (2)	0.0696 (19)	−0.0008 (18)	0.0532 (18)	0.0014 (17)
C13	0.081 (2)	0.109 (3)	0.092 (2)	−0.0239 (19)	0.055 (2)	0.0053 (19)
C12	0.087 (2)	0.085 (2)	0.116 (3)	−0.0347 (19)	0.064 (2)	−0.021 (2)
C11	0.077 (2)	0.0608 (17)	0.095 (2)	−0.0165 (14)	0.0536 (18)	−0.0173 (15)
C2	0.0450 (14)	0.0756 (19)	0.0676 (17)	0.0094 (13)	0.0120 (13)	0.0298 (14)
C9	0.0389 (13)	0.104 (2)	0.0453 (14)	−0.0153 (13)	0.0144 (11)	−0.0201 (14)
C8	0.0348 (14)	0.173 (4)	0.0450 (16)	−0.0165 (19)	0.0090 (12)	−0.020 (2)

### Geometric parameters (Å, °)

**Table:**

P1—O1	1.4803 (15)	C6—C7	1.362 (5)
P1—N2	1.6271 (19)	C7—C8	1.362 (5)
P1—N3	1.6333 (17)	C7—H7	0.9300
P1—N1	1.6808 (17)	C18—C17	1.521 (4)
O2—C1	1.216 (3)	C18—H18A	0.9700
F1—C6	1.363 (4)	C18—H18B	0.9700
N1—C1	1.368 (3)	C17—H17A	0.9700
N1—H1	0.78 (2)	C17—H17B	0.9700
C1—C4	1.490 (3)	C10—C11	1.515 (3)
C3—N3	1.467 (3)	C10—C15	1.518 (3)
C3—H3A	0.9600	C10—H10	0.9800
C3—H3B	0.9600	C15—C14	1.515 (4)
C3—H3C	0.9600	C15—H15A	0.9700
N2—C2	1.472 (3)	C15—H15B	0.9700
N2—C10	1.476 (3)	C14—C13	1.516 (4)
C19—C18	1.487 (6)	C14—H14A	0.9700
C19—C20	1.518 (7)	C14—H14B	0.9700
C19—H19A	0.9700	C13—C12	1.513 (5)
C19—H19B	0.9700	C13—H13A	0.9700
C20—C21	1.533 (5)	C13—H13B	0.9700
C20—H20A	0.9700	C12—C11	1.519 (4)
C20—H20B	0.9700	C12—H12A	0.9700
C21—C16	1.514 (4)	C12—H12B	0.9700
C21—H21A	0.9700	C11—H11A	0.9700
C21—H21B	0.9700	C11—H11B	0.9700
C16—N3	1.476 (3)	C2—H2A	0.9600
C16—C17	1.520 (4)	C2—H2B	0.9600
C16—H16	0.9800	C2—H2C	0.9600
C4—C5	1.383 (4)	C9—C8	1.386 (4)
C4—C9	1.388 (3)	C9—H9	0.9300
C5—C6	1.377 (3)	C8—H8	0.9300
C5—H5	0.9300		
			
O1—P1—N2	116.35 (10)	C19—C18—C17	111.1 (4)
O1—P1—N3	110.95 (9)	C19—C18—H18A	109.4
N2—P1—N3	105.90 (10)	C17—C18—H18A	109.4
O1—P1—N1	105.93 (9)	C19—C18—H18B	109.4
N2—P1—N1	106.50 (9)	C17—C18—H18B	109.4
N3—P1—N1	111.17 (9)	H18A—C18—H18B	108.0
C1—N1—P1	126.58 (16)	C16—C17—C18	110.9 (3)
C1—N1—H1	118.4 (17)	C16—C17—H17A	109.5
P1—N1—H1	113.4 (16)	C18—C17—H17A	109.5
O2—C1—N1	122.3 (2)	C16—C17—H17B	109.5
O2—C1—C4	121.5 (2)	C18—C17—H17B	109.5
N1—C1—C4	116.18 (19)	H17A—C17—H17B	108.0
N3—C3—H3A	109.5	N2—C10—C11	111.3 (2)
N3—C3—H3B	109.5	N2—C10—C15	114.0 (2)
H3A—C3—H3B	109.5	C11—C10—C15	111.6 (2)
N3—C3—H3C	109.5	N2—C10—H10	106.5
H3A—C3—H3C	109.5	C11—C10—H10	106.5
H3B—C3—H3C	109.5	C15—C10—H10	106.5
C2—N2—C10	117.30 (19)	C14—C15—C10	111.0 (2)
C2—N2—P1	116.26 (16)	C14—C15—H15A	109.4
C10—N2—P1	124.22 (15)	C10—C15—H15A	109.4
C18—C19—C20	110.6 (3)	C14—C15—H15B	109.4
C18—C19—H19A	109.5	C10—C15—H15B	109.4
C20—C19—H19A	109.5	H15A—C15—H15B	108.0
C18—C19—H19B	109.5	C15—C14—C13	111.6 (3)
C20—C19—H19B	109.5	C15—C14—H14A	109.3
H19A—C19—H19B	108.1	C13—C14—H14A	109.3
C19—C20—C21	112.0 (3)	C15—C14—H14B	109.3
C19—C20—H20A	109.2	C13—C14—H14B	109.3
C21—C20—H20A	109.2	H14A—C14—H14B	108.0
C19—C20—H20B	109.2	C12—C13—C14	110.4 (3)
C21—C20—H20B	109.2	C12—C13—H13A	109.6
H20A—C20—H20B	107.9	C14—C13—H13A	109.6
C16—C21—C20	109.7 (3)	C12—C13—H13B	109.6
C16—C21—H21A	109.7	C14—C13—H13B	109.6
C20—C21—H21A	109.7	H13A—C13—H13B	108.1
C16—C21—H21B	109.7	C13—C12—C11	111.3 (3)
C20—C21—H21B	109.7	C13—C12—H12A	109.4
H21A—C21—H21B	108.2	C11—C12—H12A	109.4
N3—C16—C21	112.0 (2)	C13—C12—H12B	109.4
N3—C16—C17	112.4 (2)	C11—C12—H12B	109.4
C21—C16—C17	110.9 (2)	H12A—C12—H12B	108.0
N3—C16—H16	107.1	C10—C11—C12	110.4 (3)
C21—C16—H16	107.1	C10—C11—H11A	109.6
C17—C16—H16	107.1	C12—C11—H11A	109.6
C3—N3—C16	117.01 (18)	C10—C11—H11B	109.6
C3—N3—P1	123.44 (16)	C12—C11—H11B	109.6
C16—N3—P1	118.25 (15)	H11A—C11—H11B	108.1
C5—C4—C9	120.2 (2)	N2—C2—H2A	109.5
C5—C4—C1	122.4 (2)	N2—C2—H2B	109.5
C9—C4—C1	117.4 (2)	H2A—C2—H2B	109.5
C6—C5—C4	117.8 (3)	N2—C2—H2C	109.5
C6—C5—H5	121.1	H2A—C2—H2C	109.5
C4—C5—H5	121.1	H2B—C2—H2C	109.5
C7—C6—F1	119.7 (3)	C8—C9—C4	119.2 (3)
C7—C6—C5	123.4 (3)	C8—C9—H9	120.4
F1—C6—C5	116.9 (3)	C4—C9—H9	120.4
C6—C7—C8	118.0 (3)	C7—C8—C9	121.4 (3)
C6—C7—H7	121.0	C7—C8—H8	119.3
C8—C7—H7	121.0	C9—C8—H8	119.3
			
O1—P1—N1—C1	−165.46 (18)	N1—C1—C4—C9	154.3 (2)
N2—P1—N1—C1	70.1 (2)	C9—C4—C5—C6	0.1 (3)
N3—P1—N1—C1	−44.8 (2)	C1—C4—C5—C6	−178.2 (2)
P1—N1—C1—O2	−5.7 (3)	C4—C5—C6—C7	−1.4 (4)
P1—N1—C1—C4	173.91 (15)	C4—C5—C6—F1	−179.8 (2)
O1—P1—N2—C2	60.3 (2)	F1—C6—C7—C8	179.8 (3)
N3—P1—N2—C2	−63.5 (2)	C5—C6—C7—C8	1.4 (5)
N1—P1—N2—C2	178.06 (19)	C20—C19—C18—C17	56.5 (5)
O1—P1—N2—C10	−102.28 (19)	N3—C16—C17—C18	−176.7 (3)
N3—P1—N2—C10	133.94 (18)	C21—C16—C17—C18	57.1 (4)
N1—P1—N2—C10	15.5 (2)	C19—C18—C17—C16	−57.4 (4)
C18—C19—C20—C21	−56.3 (5)	C2—N2—C10—C11	63.9 (3)
C19—C20—C21—C16	55.5 (4)	P1—N2—C10—C11	−133.7 (2)
C20—C21—C16—N3	178.0 (3)	C2—N2—C10—C15	−63.3 (3)
C20—C21—C16—C17	−55.6 (4)	P1—N2—C10—C15	99.0 (2)
C21—C16—N3—C3	58.5 (3)	N2—C10—C15—C14	−178.1 (2)
C17—C16—N3—C3	−67.1 (3)	C11—C10—C15—C14	54.8 (3)
C21—C16—N3—P1	−134.1 (2)	C10—C15—C14—C13	−55.0 (3)
C17—C16—N3—P1	100.3 (2)	C15—C14—C13—C12	56.0 (4)
O1—P1—N3—C3	−156.59 (19)	C14—C13—C12—C11	−56.9 (4)
N2—P1—N3—C3	−29.5 (2)	N2—C10—C11—C12	175.9 (3)
N1—P1—N3—C3	85.8 (2)	C15—C10—C11—C12	−55.6 (4)
O1—P1—N3—C16	36.84 (18)	C13—C12—C11—C10	56.8 (4)
N2—P1—N3—C16	163.94 (15)	C5—C4—C9—C8	1.1 (4)
N1—P1—N3—C16	−80.77 (17)	C1—C4—C9—C8	179.5 (2)
O2—C1—C4—C5	152.3 (2)	C6—C7—C8—C9	−0.1 (5)
N1—C1—C4—C5	−27.3 (3)	C4—C9—C8—C7	−1.1 (4)
O2—C1—C4—C9	−26.1 (3)		

### Hydrogen-bond geometry (Å, °)

**Table:**

*D*—H···*A*	*D*—H	H···*A*	*D*···*A*	*D*—H···*A*
N1—H1···O1^i^	0.78 (2)	2.04 (2)	2.807 (2)	165 (2)

Symmetry codes: (i) −*x*+1/2, −*y*+3/2, −*z*.

## References

[bb1] Allen, F. H., Johnson, O., Shields, G. P., Smith, B. R. & Towler, M. (2004). *J. Appl. Cryst.* **37**, 335–338.

[bb2] Bernstein, J., Davis, R. E., Shimoni, L. & Chang, N.-L. (1995). *Angew. Chem. Int. Ed. Engl.* **34**, 1555–1573.

[bb3] Bruker (2007). *APEX2* and *SAINT* Bruker AXS Inc., Madison, Wisconsin, USA.

[bb11] Dolomanov, O. V., Blake, A. J., Champness, N. R. & Schröder, M. (2003). *J. Appl. Cryst.* **36**, 1283–1284.

[bb4] Gholivand, K., Mahzouni, H. R., Pourayoubi, M. & Amiri, S. (2010). *Inorg. Chim. Acta*, **363**, 2318–2324.

[bb5] Gubina, K. E., Ovchynnikov, V. A., Swiatek-Kozlowska, J., Amirkhanov, V. M. & Domasevitch, K. V. (2002). *Polyhedron*, **21**, 963–967.

[bb6] Pourayoubi, M., Fadaei, H. & Parvez, M. (2011*a*). *Acta Cryst.* E**67**, o2046. 10.1107/S1600536811027681 PMC3213494 22091073

[bb7] Pourayoubi, M., Golen, J. A., Rostami Chaijan, M., Divjakovic, V., Negari, M. & Rheingold, A. L. (2011*b*). *Acta Cryst.* C**67**, m160–m164. 10.1107/S010827011101403X 21540535

[bb8] Pourayoubi, M. & Saneei, A. (2011). *Acta Cryst.* E**67**, o2201. 10.1107/S1600536811030194 PMC3213631 22091208

[bb9] Pourayoubi, M., Tarahhomi, A., Rheingold, A. L. & Golen, J. A. (2011*c*). *Acta Cryst.* E**67**, o934. 10.1107/S1600536811009640 PMC3100001 21754203

[bb10] Sheldrick, G. M. (2008). *Acta Cryst.* A**64**, 112–122. 10.1107/S0108767307043930 18156677

[bb12] Toghraee, M., Pourayoubi, M. & Divjakovic, V. (2011). *Polyhedron*, **30**, 1680–1690.

[bb13] Trush, V. A., Gubina, K. E., Amirkhanov, V. M., Swiatek-Kozlowska, J. & Domasevitch, K. V. (2005). *Polyhedron*, **24**, 1007–1014.

[bb14] Znovjyak, K. O., Moroz, O. V., Ovchynnikov, V. A., Sliva, T. Yu., Shishkina, S. V. & Amirkhanov, V. M. (2009). *Polyhedron*, **28**, 3731–3738.

